# Evaluation of a Highly Efficient DNA Extraction Method for *Bacillus anthracis* Endospores

**DOI:** 10.3390/microorganisms8050763

**Published:** 2020-05-20

**Authors:** Mandy Knüpfer, Peter Braun, Kathrin Baumann, Alexandra Rehn, Markus Antwerpen, Gregor Grass, Roman Wölfel

**Affiliations:** 1 Bacteriology and Toxinology, Bundeswehr Institute of Microbiology, 80937 Munich, Germany; peter3braun@bundeswehr.org (P.B.); BaumannK@rki.de (K.B.); gregorgrass@bundeswehr.org (G.G.); romanwoelfel@bundeswehr.org (R.W.); 2 Microbial Genomics and Bioinformatics, Bundeswehr Institute of Microbiology, 80937 Munich, Germany; AlexandraRehn@Bundeswehr.org (A.R.); markusantwerpen@bundeswehr.org (M.A.)

**Keywords:** *Bacillus anthracis*, spores, DNA extraction, ddPCR

## Abstract

A variety of methods have been established in order to optimize the accessibility of DNA originating from *Bacillus* *anthracis* cells and endospores to facilitate highly sensitive molecular diagnostics. However, most endospore lysis techniques have not been evaluated in respect to their quantitative proficiencies. Here, we started by systematically assessing the efficiencies of 20 DNA extraction kits for vegetative *B.* *anthracis* cells. Of these, the Epicentre MasterPure kit gave the best DNA yields and quality suitable for further genomic analysis. Yet, none of the kits tested were able to extract reasonable quantities of DNA from cores of the endospores. Thus, we developed a mechanical endospore lysis protocol, facilitating the extraction of high-quality DNA. Transmission electron microscopy or the labelling of spores with the indicator dye propidium monoazide was utilized to assess lysis efficiency. Finally, the yield and quality of genomic spore DNA were quantified by PCR and they were found to be dependent on lysis matrix composition, instrumental parameters, and the method used for subsequent DNA purification. Our final standardized lysis and DNA extraction protocol allows for the quantitative detection of low levels (<50 CFU/mL) of *B. anthracis* endospores and it is suitable for direct quantification, even under resource-limited field conditions, where culturing is not an option.

## 1. Introductions 

At the onset of extreme environmental conditions, some Gram-positive bacteria develop a very hardy resting stage: the endospore. The most prominent pathogen among these endospore-forming bacteria is *Bacillus anthracis*, a rod-shaped bacterium that causes the zoonotic disease anthrax. *Bacillus* spores show a high tenacity and persist in the environment for up to decades, surviving heat, pressure, extreme pH-values, and UV radiation [1]. The resistance to chemical and physical onslaught is due to the complex, multilayer structure of *Bacillus* spores, which features a layered cortex and a coat [2]. Spores of *B. anthracis* have been used in biological warfare programs, bioterrorism, and biocrimes in the past because of the spores’ environmental stability, as well as ease of production and dissemination [3,4]. Today, *B. anthracis* is still considered to be a relevant biological threat agent with a naturally broad geographic distribution.

The current identification methods for *B. anthracis* spores include besides molecular techniques, conventional culturing on (semi-) selective media, phage susceptibility, and biochemical or serological analysis, typically after the germination of the spores. However, specific immune-detection of *B. anthracis* is challenging, due to the high cross-reactivity of many antibodies with closely related species, such as *Bacillus cereus*, *Bacillus thuringiensis,* and other members of the *B. cereus sensu lato* (*s.l.*) group [5].

Likewise, classical microbiological methods, such as cultivation of *B. anthracis* on the semi-selective medium polymyxin-lysozyme EDTA-thallous acetate (PLET) agar, frequently fail to prevent growth of other *B. cereus s.l*. bacteria. Additionally, such tests are done at the cost of analysis time, because results take up to two days [6]. Conversely, nucleic acid-based techniques, e.g., polymerase chain reaction (PCR) amplification [7], represent a faster, more specific, and more sensitive approach for the detection of *B. anthracis*. Thus, PCR has become the standard tool for the identification of the anthrax pathogen in medical microbiology and bioforensics in the past decades [8].

As the quality of extracted nucleic acids plays a vital role for many downstream applications, such as PCR, DNA-hybridization analysis, and DNA-sequencing, the need for fast and efficient methods for DNA extraction, especially from spores, for the improved diagnosis and surveillance of microbial disease has also increased. Unlike most short-read sequencing techniques, which only require very small amounts of DNA, the extraction of inhibitor-free, high molecular weight DNA, which is suitable for long-read sequencing, has always been a challenge due to DNA fragmentation during extraction [9]. The quality of DNA extracted from clinical and environmental samples is crucial for optimum performance of these assays but numerous substances can inhibit downstream molecular applications, e.g., haemoglobin [10], polysaccharides, or humic acids [11,12]. Van Heesch et al. (2013), for instance, found that low purity of DNA negatively affects the reproducibility of sequencing-library preparations and sequencing coverage values for subsequent assemblies [13]. Therefore, commercially available DNA extraction kits are usually preferred, as these are simple and reliable and they offer excellent reproducibility and quality control [14]. However, successful DNA extraction from *B. anthracis* is highly dependent on the efficient lysis of cells and spores. Methods for cell lysis can be mainly categorized in mechanical and non-mechanical, e.g., physical, chemical, and enzymatic techniques [15]. The choice of cell lysis method depends on the type of cells (e.g., Gram-positive/Gram-negative bacteria or spores), concentration of cells, and type of matrix. Cell lysis may pose a significant challenge for thick-walled microorganisms, such as *Mycobacterium* spp. and *Bacillus* spores, and can therefore affect bacterial community profiles as well as DNA integrity [16]. No universal DNA extraction method exists that is suitable for all types of microorganisms due to the varying susceptibility of cells to lysis.

The aim of this study was to test 20 commercially available kits for the extraction of genomic DNA from vegetative cells and spores of *B. anthracis*. These kits were compared for their sensitivities, robustness, overall efficiencies, as well as utility and applicability for subsequent DNA sequencing. Notably, none of the kits tested were able to extract significant DNA from within spores. Thus, we developed and evaluated an easy-to-perform and time-efficient bead-based mechanical lysis protocol facilitating the extraction of amplifiable, high-quality DNA from within *B. anthracis* spores.

## 2. Materials and Methods

### 2.1. Strains and Culture Conditions

Attenuated *B. anthracis* Sterne (pXO1^+^, pXO2^−^) was cultivated in Brain-Heart Infusion (BHI) medium (Merck, Darmstadt, Germany) under aerobic conditions at 37 °C. The non-sporulating, Gram-negative control organism *Francisella tularensis* subsp. *holarctica* live vaccine strain (LVS) was grown in Mueller–Hinton (MHII) broth (Becton Dickinson, Heidelberg, Germany), supplemented with 2% IsoVitaleX (Becton Dickinson, Heidelberg, Germany) at 37 °C, under 5% CO_2_ atmosphere for 48 h.

### 2.2. Bacterial Standard for DNA Extraction Procedures

A mixture of defined cell numbers of vegetative *B. anthracis* Sterne and *F. tularensis* cells were used for the comparison of the different DNA extraction kits. For this, the overnight cultures of *B. anthracis* Sterne and *F. tularensis* were mixed in a ratio of 1:1, centrifuged, and the cell pellets stored at −20 °C until further use.

### 2.3. Spore Production and Purification

The sporulation of *B. anthracis* Sterne was induced by cultivation on Malvar agar, as described previously [17]. After one-week of incubation at 37 °C, the spores were harvested and a heat inactivation step (65 °C for 30 min) was performed to inactivate any remaining vegetative cells or germinating spores. Afterwards, the spores were washed three times by centrifugation at 14,000× *g* and 4 °C for 10 min, and finally resuspended in sterile distilled water. After verification of quantity and quality by phase-contrast microscopy (Leica DMi8, Leica, Wetzlar, Germany), spores harbouring less than 1% vegetative cells and debris were stored at concentrations of approximately 10^9^ CFU/mL at 4 °C for a maximum of 12 months without measurable decline of viability.

### 2.4. DNA Extraction

#### 2.4.1. Extraction of DNA Using Commercial Kits

Equal amounts of starting material were used to enable direct comparison of the kits’ efficacy. For this, the bacterial standard consisting of a defined number of vegetative cells of *B. anthracis* and non-spore-forming *F. tularensis* (as a process control) was used. DNA was extracted in triplicate when using 20 different commercial kits (Table 1), according to their respective manufacturers’ instructions. If available, a DNA extraction protocol for Gram-positive bacteria was used. Sterile water was processed alongside and it served as extraction control to monitor cross contamination. Extracted DNA was stored at −20 °C until further use.

#### 2.4.2. Modified DNA Extraction Protocol for the MasterPure Complete DNA and RNA Purification Kit

Bacterial cell pellets (10^1^ to 10^10^ cells) were resuspended in 150 µL TE buffer containing 1250 U of Ready-Lyse Lysozyme Solution (Lucigen, Middleton, WI, USA) and then incubated at 37 °C for 60 min. Subsequently, 150 μL of Tissue and Cell Lysis Solution containing 1 μL of Proteinase K (50 μg/μL) were added. The mixture was incubated for 15 min with shaking at 900 rpm at 65 °C, and vortexed every 5 min. After placing the samples on ice for 5 min, 175 µL of MPC Protein Precipitation Reagent were added. Cell debris was pelleted by centrifugation at 4 °C for 10 min at 13,100× *g* in a microcentrifuge. The supernatant was transferred to a fresh microcentrifuge tube and the pellet was discarded. A volume of 5 µL Roti-Pink (Carl Roth, Karlsruhe, Germany), 10 µL glycogen solution (5 mg/mL, Carl Roth, Karlsruhe, Germany), and 500 µL isopropanol (Carl Roth, Karlsruhe, Germany) were added to the recovered supernatant and then carefully mixed by inverting the tube several times. The precipitated DNA was pelleted by centrifugation at 4 °C for 10 min at 13,100× *g* in a microcentrifuge. After washing DNA pellets twice with 200 µL of 70% ethanol, the DNA was resuspended in 50 µL TE (10 mM Tris-HCl [pH 7.5], 1 mM EDTA) buffer and then stored at −20 °C until further use.

### 2.5. qPCR Assay

Table 2 summarizes the oligonucleotides used in this study and they were synthesized by TIB Molbiol (Berlin, Germany). For the detection of *B. anthracis*, the chromosomal marker *dhp*61 was used as described previously [18].

DNA of *F. tularensis* was detected by targeting the 16S rRNA gene. Briefly, a 20 μL amplification mixture contained 2 μL LightCycler FastStart DNA Master HybProbe (Roche Diagnostics, Mannheim, Germany), 4 mM MgCl_2_, 1 μM primer 16S Fran_F, 1 μM primer Fran_R, 1 μM probe, and 5 μL of extracted genomic DNA. qPCR was performed on a LightCycler 480 II instrument (Roche diagnostics, Mannheim, Germany) at 95 °C for 10 min and 45 cycles of denaturation at 95 °C for 20 s, annealing at 58 °C for 20 s, and extension at 72 °C for 30 s. The quantification cycle values were automatically calculated by the LightCycler 480 software version 1.5.0.39 using the second derivative method. All of the qPCR assays were performed in triplicate.

### 2.6. DNA Yield and Quality

Concentrations of extracted DNAs were assessed using the Qubit 2.0 fluorometer (Invitrogen, Thermo Fisher Scientific, Darmstadt, Germany). Briefly, 10 µL of extracted genomic DNA was mixed with 190 µL of a Qubit working solution (Qubit High Sensitivity Assay, Invitrogen, Thermo Fisher Scientific, Darmstadt, Germany), according to the manufacturer’s protocol. The total DNA yield was calculated based on the DNA concentration taken from Qubit measurements and the final elution volume of the DNA extracts. Quality and purity (absorbance ratio at 260/280) of extracted genomic DNA were determined using a DS-11 FX spectrophotometer (DeNovix Inc., Wilmington, NC, USA), where pure DNA is defined as having a 260/280 absorbance ratio ranging between 1.7 and 2.0.

### 2.7. DNA Integrity

The integrity of DNA extracted by each method was assessed by gel electrophoresis. Specifically, 5 µL of each DNA extract was analyzed on a 1% agarose gel containing 1 × SYBR Gold Nucleic Acid Gel Stain (Thermo Fisher Scientific, Dreieich, Germany) and was visualized by ChemiDoc Imaging System (Bio-Rad Laboratories, Munich, Germany). Furthermore, selected samples were examined using the 5200 Fragment Analyzer system (Agilent Technologies Inc., Waldbronn, Germany). For this, the HS Genomic DNA 50 kb kit was used according to the manufacturer’s instructions and signals were analyzed using the Fragment Analyzer Software Pro Size Version 3.0.1.6 (Agilent Technologies Inc., Waldbronn, Germany).

### 2.8. Extraction of DNA from B. anthracis Spores

An additional mechanical disruption step was applied due to the multilayered structure of *B. anthracis* spores. For this purpose, 200 mg of PowerBeads (0.1 mm, Qiagen, Hilden, Germany) were added to 1 mL of a *B. anthracis* spore suspension (10^2^ to 10^8^ spores/mL) and lysis occurred for 5 min at 30 Hz in a TissueLyser II instrument (Qiagen, Hilden, Germany). After mechanical disruption, the spore suspensions were centrifuged for 10 min at 13,100× *g*. Supernatants were concentrated to a final volume of 150 µL using Microcon centrifugal filters (Merck, Darmstadt, Germany) and used for DNA extraction using the MasterPure Complete DNA and RNA Purification kit according to the manufacturer’s instructions for liquid samples. Additionally, cell pellets that were obtained by centrifugation after the bead-beating step were used for DNA extraction applying the modified protocol of the MasterPure Complete DNA and RNA Purification kit. DNA extracted from both, the cell pellet and the supernatant, were separately dissolved in 25 µL TE buffer, pooled, and then stored at −20 °C until further use.

### 2.9. Spore Treatment with Propidium Monoazide (PMA)

PMA is a photoreactive DNA-binding dye that is used to monitor cell viability (live/dead differentiation) [19]. PMA can be used to detect spores with compromised spore coat, cortex, and membranes via fluorescence microscopy because of its fluorescent properties [20]; thus, *B. anthracis* spores were treated with PMA to distinguish viable from nonviable spores. For this, the Blu-V Viability PMA kit (Qiagen, Hilden, Germany) was used according to the manufacturer’s instructions. Briefly, 10^8^ spores were centrifuged for 5 min at 13,100× *g* and then dissolved in 500 µL buffer EB. PMA was added to the samples at a final concentration of 20 µM and the mixture was stored in the dark for 10 min at room temperature. After incubation, the dye was cross-linked to DNA by exposing the samples to blue light (λ = 464−476 nm nm) for 10 min The spores were recovered by centrifugation (13,100× *g*, 10 min) and then subjected to confocal laser scanning microscopy.

### 2.10. Spore Microscopy

The PMA-stained spores were observed by confocal laser scanning microscopy (LSM 710, Zeiss, Oberkochen, Germany) at λ_Ex_ = 510 nm and λ_Em_ = 610 nm. Spore preparations were placed in an imaging chamber (µ-slide 8 well, Ibidi, Martinsried, Germany) and then covered with agarose slides [1% (*w*/*v*)] to ensure proper scanning. Fluorescence signals of covalent PMA/DNA-adducts following photolysis were recorded and merged with matching bright field images. The percentage of stained and unstained spores was determined after counting a minimum of 500 randomly selected spores.

Transmission electron microscopy (TEM) was performed on a Libra 120 (Zeiss, Oberkochen, Germany). For this, spore suspensions of *B. anthracis* were inactivated by chemical fixation in 8% formaldehyde and 2% glutaraldehyde in 0.1 M PIPES buffer for at least 4 h at room temperature. The grids were coated with alcian blue (1% in 1% acetic acids, Carl Roth, Karlsruhe, Germany), as described previously [21], and washed with distilled water. A volume of 10 µL of *B. anthracis* spore suspension was applied onto the grid and incubated for 10 min. After washing the grid surface with distilled water, the grid was placed on a drop of 0.5% uranyl acetate for 10 s, and finally dried using filter paper [22].

### 2.11. Copy Number Analysis Using Digital Droplet PCR

Digital droplet PCR (in the following abbreviated as ddPCR) used in this study to quantify the genomic DNA from *B. anthracis* spores is based on a previous qPCR assay on the chromosomal *dhp61* marker [18]. The 20 µL of the PCR mixture consisted of 10 µL ddPCR Supermix for Probes (no dUTP) master mix (Bio-Rad Laboratories, Munich, Germany), forward and reverse primer at 0.9 µM each, probe at 0.25 µM and 2 µL template DNA. PCR amplification was performed in a Mastercycler Gradient (Eppendorf, Hamburg, Germany) using the following cycling conditions: An initial denaturation step at 95 °C for 10 min, followed by 40 cycles consisting of a denaturation step at 94 °C for 30 s and an annealing-extension step at 58 °C for 1 min with a final extension at 98 °C for 10 min and an indefinite hold at 4 °C. All of the steps were carried out using a ramp rate of 2 °C/s. After cycling, droplets were immediately analyzed on the QX200™ Droplet Reader (Bio-Rad Laboratories, Munich, Germany). The data were analyzed using the software QuantaSoft^TM^ analysis pro 1.0.596 (Bio-Rad Laboratories, Munich, Germany), where thresholds were manually set for each sample.

### 2.12. Whole Genome Sequencing

Whole Genome Sequencing was performed on the Oxford Nanopore Technologies (ONT) GridION platform (Oxford Nanopore Technologies Ltd, Oxford, UK). For this, library preparation was conducted, according to the manufacturers’ protocol using the Native Barcoding expansion pack (EXP-NBD104) and the 1D Sequencing kit, with the SQK-LSK109 chemistry (both Oxford Nanopore Technologies Ltd, Oxford, United Kingdom). No additional shearing was performed and the DNA repair step was done according to the protocol, starting with 100 ng of DNA per sample. Libraries were sequenced on R9.4 flow cells and reads were collected as fastq-reads using GridION-software (Release 19.12.2, Oxford Nanopore Technologies Ltd, Oxford, United Kingdom). The software NanoPlot 1.24.0 (Available online: https://github.com/wdecoster/NanoPlot) was used without any filter-options for investigation and visualization of quality and length of obtained sequencing-reads.

## 3. Results

### 3.1. Comparison of DNA Extraction Kits

The extraction of high quality DNA in sufficient quantity is essential for genome sequencing. The goal of this study was to evaluate and compare different commercially available kits for DNA extraction from vegetative cells and spores of *B. anthracis* to address this requirement. Since extraction efficiencies may differ between Gram-positive and Gram-negative bacteria due to their peptidoglycan structure, we included *F. tularensis* as representative for Gram-negative species in our study.

#### 3.1.1. Characteristics of DNA Extraction Kits

The kits tested in this study rely on different principles for DNA purification, including solid-phase extraction techniques (e.g., anion-exchange methods, silica-membrane technology, and magnetic-particle technology) and solution-based (salting-out) protocols, which are based on the precipitation of DNA. Table 3 lists the basic characteristics of the kits compared in this study. The most rapid extraction method was the NucleoSpin Microbial DNA kit, while the most time-consuming were the Wizard Genomic DNA Purification kit, the QIAamp Cador pathogen Mini kit and the in-house developed protocol. However, most of the required time did not involve hands-on activities (e.g., longer incubation times). The average extraction costs per sample varied from 2.23 to 16.97 €, with salting-out kits being the least expensive.

#### 3.1.2. DNA Extraction Method Greatly Impacts DNA Yield

**Figure 1:**
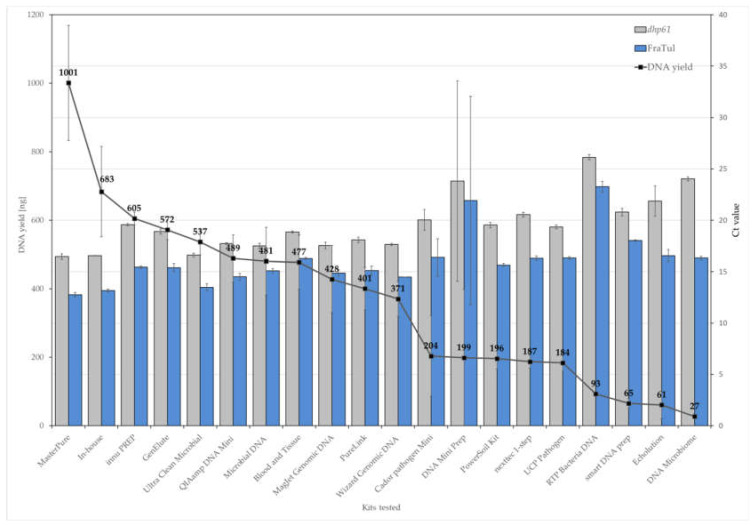
DNA extraction efficiency depends on the method (kit) used. Average DNA yields (black line) and qPCR amplification results of samples containing *B. anthracis* (*dhp*61, grey columns) or *F. tularensis* (16S FraTul, blue columns) cells, obtained with the following 20 commercial kits: (1) MasterPure Complete DNA and RNA Purification kit; (2) In-house protocol, based on the DNA Investiagtor kit; (3) innu PREP DNA Mini kit; (4) GenElute Bacterial Genomic DNA kit; (5) DNeasy Ultra Clean Microbial kit; (6) QIAamp DNA Mini kit; (7) NucleoSpin Microbial DNA Mini kit; (8) DNeasy Blood and Tissue kit; (9) MagJet Genomic DNA kit; (10) PureLink Microbiome DNA Purification kit; (11) Wizard Genomic DNA Purification kit; (12) QIAamp Cador pathogen Mini kit; (13) DNA Mini Prep; (14) DNeasy PowerSoil kit; (15) nexttec 1-step DNA isolation kit for Bacteria; (16) QIAamp UCP Pathogen Mini kit; (17) RTP Bacteria DNA Mini kit; (18) smart DNA prep; (19) Echolution Tissue DNA Micro kit; and, (20) QIAmp DNA Microbiome kit. Data are presented as the mean of triplicates and the error bars show standard deviations.

Total DNA yield varied significantly, depending on the DNA extraction method used (Figure 1 and Supplementary Material, Table S1). The MasterPure Complete DNA and RNA Purification kit (hereinafter referred to as MasterPure kit) extracted the highest quantity of DNA, followed by the in-house protocol, and the innu PREP DNA Mini kit from Analytik Jena, yet the MasterPure kit extracted significantly more DNA when compared to all other protocols used in the present study. These results were also confirmed by qPCR, which was used to evaluate the presence of amplifiable DNA and PCR inhibitors in the extracts. All of the tested methods successfully extracted bacterial DNA from both *B. anthracis* and *F. tularensis*, but with varying efficiencies, as demonstrated by genus-specific PCR (Figure 1 and Supplementary Material, Table S1). The MasterPure kit gave the highest DNA recovery (lowest C_t_ values, Figure 1) for both organisms, followed by the in-house protocol and the DNeasy Ultra Clean Microbial kit. Negative extraction controls (nuclease-free water) for all kits yielded no qPCR signals.

The purity of extracted DNA was spectrophotometrically determined. While DNA extracted with the MasterPure kit and the in-house protocol met the set A260/A280 absorbance ratio (1.8–2.0) recommended for downstream applications, other kits deviated negatively from this range. Furthermore, significant differences in 260/230 absorbance ratios were detected among DNA extraction methods, which reflected the co-extraction of contaminants absorbing at 230 nm, such as residual guanidine HCl from nucleic acid extraction (Supplementary Material, Table S1 and Figure S1).

The integrity of the extracted DNA was assessed by agarose gel and capillary electrophoresis. Most tested kits yielded high-molecular-weight genomic DNA. However, DNA extracted with the smart DNA prep kit and the QIAmp DNA Microbiome kit showed no distinct bands on agarose gels, which is probably due to low DNA concentrations (see Supplementary Material, Figure S2). DNA extracted with RTP Bacteria DNA Mini kit only resulted in significantly sheared DNA with an average fragment size of 500–1000 bp (Figure S2). Differences in fragment lengths of DNA isolated using different kits were also confirmed using the Fragment Analyzer system, showing that the MasterPure kit provided the largest DNA fragments of up to 100 kb (Supplementary Material, Figure S3).

#### 3.1.3. An Improved DNA Extraction for Superior DNA Yields

DNA extracted with the MasterPure kit was found to contain large amounts of RNA when analyzed by gel electrophoresis. After the addition of RNase A, DNA yields were unaffected (925 ± 38 ng with RNAse treatment and 1001 ± 168 ng without RNAse treatment). Although this kit proved to be the best DNA extraction kit for both bacteria, *B. anthracis* and *F. tularensis*, the kit does not include a lysozyme lysis step. Therefore, the standard protocol was extended by a lysozyme treatment. The usage of lysozyme is recommended in many protocols for the pre-treatment of Gram-positive bacteria in order to digest the thick peptidoglycan layer to increase the total DNA yield and improve species representation in microbiota composition studies [23,24,25,26]. Indeed, the use of lysozyme for the MasterPure kit increased DNA yields from 469 ± 8 ng to 573 ± 11 ng (per 10^7^ cells), which also correlated with a decrease of the C_t_ values of *dhp*61 qPCR (from 17.60 ± 0.01 to 16.05 ± 0.05).

Glycogen and Roti^®^PinkDNA are known precipitation additives that are used to increase nucleic acid recovery from alcohol precipitation [27]. Thus, we compared DNA recovery efficiencies in the presence or absence of glycogen and Roti^®^PinkDNA. Neither of the additives had any negative effect on DNA integrity; high-molecular weight DNA could be obtained from all combinations (Supplementary Material, Figure S4). The addition of either glycogen or Roti^®^PinkDNA resulted in only slightly higher DNA concentrations, as can be observed from the slightly increased RFU values in the Fragment Analyzer system. However, the addition of both glycogen and Roti^®^PinkDNA resulted in a striking increase of the DNA concentration, as shown by ddPCR (Supplementary Material, Table S2). Furthermore, the glycogen/Roti^®^PinkDNA/nucleic acid precipitate forms a visible pellet, which simplifies downstream sample processing and leads to a higher reproducibility. Therefore, the final DNA extraction protocol using the MasterPure Complete DNA and RNA Purification kit was extended by the addition of both, glycogen and Roti^®^PinkDNA.

Next, the DNA extraction efficiency of the MasterPure kit was assessed by qPCR. The sensitivities were determined as the minimum of cells of *B. anthracis* or *F. tularensis* that led to a positive result in qPCR assays. This approach allowed for the reproducible detection of *B. anthracis* at 8.6 × 10^1^ and *F. tularensis* at 5.0 × 10^1^ cells (Supplementary Material, Table S3).

Next, we compared the MasterPure kit and the QIAamp DNA Mini kit regarding their ability to deal with an overload of starting material. For this purpose, the *B. anthracis* cells were spiked in (A) an overnight culture of *E. coli* (approximately 10^10^ cells) and (B) in 1 mL of sheep blood. Both of the methods recovered *B. anthracis* DNA in the presence of high background DNA concentrations, as shown by qPCR (Supplementary Material, Table S4). Yet, the MasterPure kit provided a significant higher DNA extraction yield from either matrix. These results indicate that the salting-out method (MasterPure kit) was more efficacious in recovering target DNA under high concentrations of non-target DNA and that the type of matrix used had a strong effect on the amount of total and amplifiable DNA recovered.

#### 3.1.4. DNA Extraction Method Impacts the Quality of Sequencing Results

Improving the efficiency of DNA extraction from complex biological or environmental samples is a highly desirable goal [28,29], especially for genome sequencing technologies, such as long-read sequencing technologies, like nanopore sequencing [9,30]. Thus, we constructed libraries from genomic DNA of *B. anthracis* isolated with the QIAamp DNA Mini kit or with the MasterPure kit to quantify the influence of the extracted DNA on the quality of the sequencing reads. The quality of the sequencing data was analyzed in read-lengths vs read-qualities plots. In this context, the quality score estimates the probability that a given base position is incorrectly sequenced, for example a quality score of 10 represents an error rate of 1 in 10, with a corresponding accuracy of 90%. This quality score can be influenced, among others, by the quality of DNA samples as high quality DNA samples generate larger data with a higher quality score [31]. DNA generated with the QIAamp kit showed an average read length of 500 bp and a low quality score of 4–7. In contrast, DNA extracted with the MasterPure kit showed a read quality of 11 and the average read length generated during the GridION run was about 10,000 bp (Supplementary Material, Figure S5).

Taken together, we showed that DNA yield, purity, and integrity were the highest for the test organisms *B. anthracis* and *F. tularensis* when using the MasterPure Complete DNA and RNA Purification kit supplemented with RNAse, glycogen, and tracing-dye Roti^®^PinkDNA.

### 3.2. DNA Can Be Effectively Extracted from Within B. anthracis Spores

#### 3.2.1. Commercial DNA Extraction Kits Are Not Suitable to Lyse Spores

**Figure 2:**
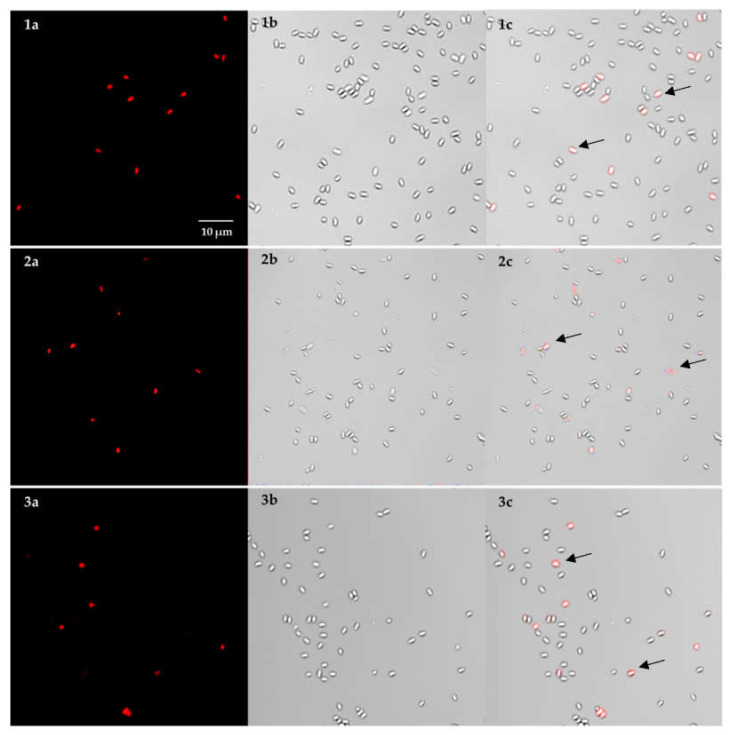
Confocal laser scanning microscopy images of *B. anthracis* spores pre-treated with PMA before and after DNA extraction by two different methods. Micrographs were taken before (**1**) and after DNA extraction with the MasterPure Complete DNA and RNA Purification kit (**2**) and the QIAamp DNA Mini kit as comparison (**3**), respectively; (**a**) red channel (PMA dyed) to detect spores with compromised spore coat, cortex and membranes; (**b**) Bright field image, (**c**) Overlay of bright field and PMA signal; Scale bar: 10 μm. Arrows indicate spores with compromised spore coat, cortex, and membranes.

Next, we took on the challenge of efficiently extracting DNA from within spores of *B. anthracis*. Since the MasterPure kit was ranked first for DNA extraction from both Gram-positive and Gram-negative bacteria, it was also tested on a suspension of *B. anthracis* spores. Alongside, we also tested the QIAamp DNA Mini kit, as it is commonly used for DNA extraction in laboratories [32]. Of note, there is residual DNA of the spore mother cell remaining on the spore surface, which, at least in part, is accessible for PCR [33,34,35]. Nevertheless, retrieved DNA concentrations were not sufficient for further downstream applications, such as nanopore sequencing, since library preparation requires a minimum of 0.5–1 µg of DNA. We suspected that both kits were able to only extract this surface-adsorbed DNA without efficaciously lysing spores and, thus, gain access to DNA contained within the spore core. Of note, the MasterPure kit contains a centrifugation step during which the cell debris is removed. We suspected that this step could be responsible for the loss of still intact spores. Therefore, the cell debris precipitate was microscopically examined for the presence of intact spores using the cell (and spore) impermeable fluorescent dye PMA. The percentages of intact, non-fluorescent spores did not significantly differ between samples before (85%) and after (80%) treatment with the MasterPure kit or standard lysis using the QIAamp DNA Mini kit, respectively, as shown in Figure 2. These results indicate that both DNA extraction kits were not able to properly lyse spores and extract intracellular DNA. Likely only surface-attached DNA was recovered during the DNA extraction process. Thus, there is a need for a more efficient method for DNA extraction from within *B. anthracis* spores.

#### 3.2.2. Bead-Beating Enables Effective Lysis of *B. anthracis* Spores for Subsequent DNA Extraction

Previous studies have shown that the bead-beating method is the most promising method for extracting genomic DNA from *Bacillus* spp. spores [36]. Thus, we added a bead-beating step to DNA extraction and used qPCR as an indicator of lysis efficiency. First, the disruption efficiencies of the TissueLyser II and the FastPrep-24 instruments were compared. Spores were also analyzed by PCR without any mechanical disruption step as a control and to monitor the degree by which the PCR experiments predominantly detected spore-attached DNA. These unprocessed spores amplified approximately 8 to 10 C_t_ units later than samples that underwent mechanical lysis. In general, all of the samples subjected to disruption yielded more amplifiable DNA than those without (Table 4). Furthermore, in both groups of experiments, the C_t_ values of spores lysed using either TissueLyser II or FastPrep-24 were similar, indicating similar lysis efficiency. The lysis efficiency only increased slightly when treatment by the TissueLyser II was increased from 1 to 5 min. Conversely, further increasing of the processing time to 10 min reduced detectable DNA (Table 4), which might be due to concomitant DNA shearing [37,38].

Bead-beating conditions were next optimized with the aim of obtaining the largest amount of DNA with the smallest degree of DNA shearing. Since it has been shown that the disruption efficiencies of bead-beating are mainly dependent upon the composition of the lysis matrix, including size, material, and relative content of microbeads [39], we tested the impact of different beads on spore lysis efficiency. Significant differences in DNA extraction efficiencies were detected when different beads were compared (Table 4), with the PowerBead Tubes (0.1 mm) found to be superior to the other beads tested.

It has been postulated that the beads-to-cells ratio will also determine the cell disruption efficiency [40]. Therefore, we next tested this parameter on lysis efficiency. When the amounts of beads were changed, the qPCR-detectable DNA also changed. The DNA extraction efficiency was the highest when 200 mg of beads were used. The sample volume also had great influence on the DNA extraction efficiency with 1 mL sample volume leading to the lowest C_t_ values (Table 4).

**Figure 3:**
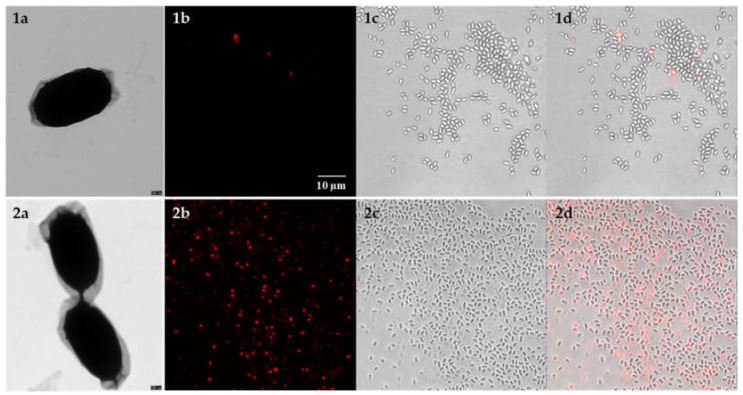
Disruption of *B. anthracis* spores after bead-beating observed by TEM and fluorescence microscopy. Micrographs show untreated spores (**1**) and spores after bead-beating (**2**); (**a**) TEM of negatively stained *B. anthracis* Sterne spores (Scale bar: 100 nm); confocal laser scanning microscopy images of *B. anthracis* spores pre-treated with PMA before (**1b**–**d**) and after (**2b**–**d**) mechanical disruption of spores by bead-beating; (**b**) red channel (PMA dyed) to detect spores with compromised spore coat, cortex and membranes; (**c**) bright field image, (**d**) Overlay of bright field and PMA signal (scale bar for all light micrographs: 10 μm).

Transmission electron microscopy (TEM) was used to visualize the damage of mechanical bead-beating on *B. anthracis* spores. Surprisingly, no visible structural changes and obvious damage to the spores and the exosporium was observed after mechanical disruption as compared to the untreated controls (Figure 3; panels 1a and 2a). The fluorescent dye PMA was used as a marker for microscopy before (1) and after (2) mechanical disruption of the spores in order to quantitatively score lysis efficiency by bead-beating. In the control (untreated spores), the percentage of red-fluorescent (i.e., damaged) spores was about 5% (compare Figure 2). The percentage of red-fluorescent spores increased to approximately 95% after mechanical disruption with bead-beating. These results strongly indicate the presence of disrupted spores and showed that bead-beating is a highly efficient method able to release intracellular amplifiable DNA from *B. anthracis* spores.

The amount of qPCR-amplifiable DNA increased approximately 100- to 1000-fold following bead-beating treatment. The largest amount of DNA was recovered using 200 mg of PowerBead Tubes (0.1 mm) and a sample volume of 1 mL in combination with the TissueLyser II (5 min at 30 Hz).

#### 3.2.3. An Improved Protocol for Highest Efficiency of DNA Extraction from within *B. anthracis* Spores

Finally, the efficiencies of different DNA extraction methods were compared in combination with bead-beating. A *B. anthracis* spore suspension with a concentration of 10^6^ spores/mL was lysed by beat-beating, as described above and two kits (the MasterPure kit and the in-house protocol, based on the DNA Investigator kit) that had performed best in DNA extraction from vegetative cells of *B. anthracis* were tested alongside on these damaged spores. For comparison, the “standard” QIAamp DNA Mini kit was also included and the extracted DNAs were quantified by qPCR and ddPCR.

For all tested kits, the mean C_t_ value for DNA extracted from mechanically disrupted spores was significantly lower than the mean C_t_ value of the untreated samples. However, large differences in DNA extraction efficiencies could be observed when comparing the different kits (Table 5) with the QIAamp DNA Mini kit yielding the highest and the in-house protocol the lowest C_t_ values. DNA extractions performed with the MasterPure kit resulted in about one unit lower C_t_ values as the in-house protocol.

The debris pellet obtained by centrifugation after the bead-beating step was also used for DNA extraction with the MasterPure kit to further increase DNA yield. Interestingly, the qPCR results suggested that very similar DNA yields could be retrieved from cell debris and from the lysis supernatant. These results were confirmed by ddPCR (see Table S5). Thus, the final protocol for the DNA extraction from *B. anthracis* spores was extended to include both, lysis supernatant and spore debris and DNA extracted was each dissolved in 25 µL TE buffer and pooled. In the end, this procedure was more efficient than the in-house protocol (Table 5).

**Figure 4:**
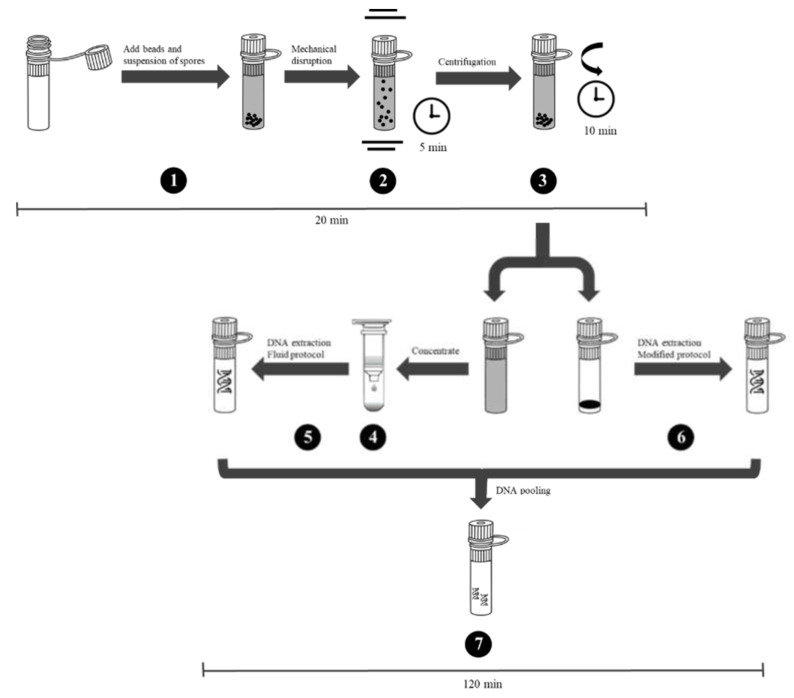
Procedure for efficient extraction of DNA from within *B. anthracis* spores. Individual experimental steps and respective time frames are indicated: (1) 1 mL of a spore suspension is added to 200 mg of PowerBead Tubes (0.1 mm); (2) tubes are processed for 5 min at 30 Hz in a TissueLyser II instrument; (3) the spore suspension is centrifuged for 10 min at 13,100× *g*; (4) the supernatant is concentrated using Microcon centrifugal filters to a final volume of 150 µL; (5) the concentrate is used for DNA extraction with the MasterPure kit (standard manufacturer’s instructions for fluid samples); (6) additionally, the debris pellet from the centrifugation step (3) is used for DNA extraction with the modified (see text for details) MasterPure protocol; (7) DNA extracted from the debris pellet and DNA isolated from lysis supernatant are each dissolved in 25 µL TE buffer and pooled.

Lastly, the efficiencies for the DNA extraction methods from within *B. anthracis* spores was determined with qPCR by log-diluting spores (one to 10^8^ spores per mL) and treating these samples for 5 min by a TissueLyser II instrument (as above). DNA was extracted using the procedure visualized in Figure 4. The limit of detection (LOD) at 0.95 probability for a positive response was calculated by probit analysis as 33 spores per mL, which corresponds to three copies per PCR-reaction [41].

## 4. Discussion

In the event of infection with an unknown pathogen, rapid detection and identification is crucial for effective treatment and containment. Despite the large number of methods, assays, and instrumentation used for the detection of *B. anthracis*, classical bacteriology, e.g., cultivation and microscopy, remains a standard for diagnosis, even though this requires at least 24 h to complete. In contrast, molecular approaches are faster and have a higher sensitivity and specificity. Therefore, qPCR has been widely used for the detection of pathogens in clinical and environmental samples and in a large number of laboratories and institutions, DNA is usually extracted with the QIAamp DNA Mini kit or the QIAamp Blood and Tissue kit [42]. However, DNA extraction efficiency is dependent on several factors e.g., the type of bacterial species and the cell concentration that is being subjected to extraction [32]. At low concentrations, method sensitivity plays a pivotal role and, at high concentrations, overloading of the method can reduce DNA extraction efficiency [43]. Since *B. anthracis* forms endospores that are resilient to many lysis buffers, several chemicals, heat and mechanical force, it is very difficult to extract DNA from their spore core [44]. A rapid-viability PCR (RV-PCR) has been developed [45], which uses a broth enrichment step to germinate *B. anthracis* spores into the easier-to-lyse vegetative cells, thus improving the detection limit to 400 CFU/mL, in order to ameliorate this issue [46]. However, this germination step increases the analysis time to at least 17 h and it is only applicable to viable spores.

Here, we tested 20 commercial DNA extraction kits for their suitability for isolating DNA from vegetative cells and spores of *B. anthracis* without a prior enrichment step. Significant differences in DNA yields and quantities were observed among these kits, with the MasterPure Complete DNA and RNA Purification kit being the most efficient for effective DNA isolation from low amounts of vegetative cells of *B. anthracis*. However, this kit was unable to isolate DNA from within *B. anthracis* spores. Various methods have been used in order to optimize the accessibility of *Bacillus* spore DNA to increase the sensitivities of diagnostic DNA-based methods, like PCR. This includes heat treatment [33,47,48,49], chemical agents, such as detergents and chaotropic salts [50,51], and mechanical disruption, e.g., sonication and bead-beating [48,52]. For example, the lysis method described by Luna et al. (2003) involving heat shock followed by sonication and autoclaving, is labour intensive and time-consuming [48]. In contrast, the simple bead-based mechanical lysis protocol developed here facilitates extracting amplifiable, high-quality DNA from the cores of *B. anthracis* spores within a reasonably short time. The final standardized lysis and DNA extraction protocol allows for the quantitative detection of low levels (<50 CFU/mL) of *B. anthracis* spores within 2.5 h and it is suitable for the direct quantification of *B. anthracis* spores, even under field conditions, when culturing is not an option.

In conclusion, this optimized DNA extraction protocol ensures efficient spore lysis, minimal DNA shearing and the removal of PCR inhibitors. It even allows for the detection of non-viable *B. anthracis* spores and the DNA is additionally compatible with GridION nanopore sequencing technology. We believe that this method can also be adapted for other clinical and environmental samples, in which hard-to-lyse organisms are often present in low numbers and for which high-efficiency DNA extraction is needed to increase the likelihood of correct pathogen identification.

## Figures and Tables

**Table 1:** Commercial DNA extraction and purification kits used in this study.

Commercial Kit	Manufacturer	Cell Disruption Method *
DNA MinipPrep kit	ZymoBIOMICS	BB/ SB
DNeasy Blood and Tissue kit	Qiagen	PL + CL/ SB
DNeasy PowerSoil kit	Qiagen	BB/ SB
DNeasy Ultra Clean Microbial kit	Qiagen	BB/ SB
Echolution Tissue DNA Micro kit	BioECHO	PL + CL/ SB
GenElute Bacterial Genomic DNA kit	Sigma Aldrich	PL + CL/ SB
In-house protocol, based on DNA Investigator kit	Qiagen	BB/ SB
innu PREP DNA Mini kit	Analytik Jena	PL + CL/ SB
MagJet Genomic DNA kit	Thermo Scientific	PL + CL/ MP
MasterPure Complete DNA & RNA Purification kit	Lucigen	PL + CL/ SP
nexttec 1-step DNA isolation kit for Bacteria	Biozym	PL + CL/ SB
NucleoSpin Microbial DNA Mini kit	Machery & Nagel	BB/ SB
PureLink Microbiome DNA Purification kit	Thermo Scientific	BB/ SB
QIAamp Cador pathogen Mini kit	Qiagen	PL + CL/ SB
QIAamp DNA Mini kit	Qiagen	PL + CL/ SB
QIAamp UCP Pathogen Mini kit	Qiagen	BB/ SB
QIAmp DNA Microbiome kit	Qiagen	BB/ SB
RTP Bacteria DNA Mini kit	Stratec Molecular	PL + CL/ SB
smart DNA prep	Analytik Jena	CL/ MP
Wizard Genomic DNA Purification kit	Promega	PL + CL/ SP

* PL physical lysis; CL chemical lysis; BB bead-beating; MP magnetic particle based technology; SB silica based technology; SP salt precipitation.

**Table 2:** Primers and probes used for qPCR amplification.

Name	Sequence [5–3′]	Reference
dhp61_183-113F	CGTAAGGACAATAAAAGCCGTTGT	[18]
dhp61_183-208R	CGATACAGACATTTATTGGGAACTACAC	[18]
dhp61_183-143T	6FAM-TGCAATCGATGAGCTAATGAACAATGACCCT-TMR	[18]
Fran_F	GAGCGCAACCCCTATTGATA	this study
Fran_R	TTTTTGAGTTTCGCTCCAGCT	this study
Fran_TM	6FAM-CTATTGAGACTGCCGCTGACAAGGC-BBQ	this study

**Table 3:** Characteristics of the 20 commercial DNA extraction kits compared.

No.	Commercial Kit	Cell Disruption Method *	Completion Time ** [min]	Average Cost *** [€/Sample]
1	MasterPure Complete DNA and RNA Purification kit	PL + CL/SP	90	2.23
2	In-house protocol, based on DNA Investigator kit	BB/ SB	180	6.13
3	innu PREP DNA Mini kit	PL + CL/SB	90	2.44
4	GenElute Bacterial Genomic DNA kit	PL + CL/SB	90	3.08
5	DNeasy Ultra Clean Microbial kit	BB/ SB	50	2.79
6	QIAamp DNA Mini kit	PL + CL/SB	150	3.90
7	NucleoSpin Microbial DNA	BB/ SB	40	3.50
8	DNeasy Blood and Tissue kit	PL + CL/SB	90	3.74
9	MagJet Genomic DNA kit	PL + CL/MP	150	3.06
10	PureLink Microbiome DNA Purification kit	BB/SB	60	6.64
11	Wizard Genomic DNA Purification kit	PL + CL/SP	180	2.49
12	QIAamp Cador pathogen Mini kit	PL + CL/SB	180	6.24
13	DNA Mini Prep	BB/SB	60	6.52
14	DNeasy PowerSoil kit	BB/SB	50	6.43
15	nexttec 1-step DNA isolation kit for Bacteria	PL + CL/SB	60	2.69
16	QIAamp UCP Pathogen Mini kit	BB/SB	60	4.86
17	RTP Bacteria DNA Mini kit	PL + CL/SB	75	3.89
18	smart DNA prep	CL/MP	150	3.40
19	Echolution Tissue DNA Micro kit	PL + CL/SB	60	3.42
20	QIAmp DNA Microbiome kit	BB/ SB	160	16.97

* PL physical lysis; CL chemical lysis; BB bead-beating; MP magnetic particle based technology; SB silica based technology; SP salt precipitation; ** Approximate time to complete DNA extraction from four samples; *** List price on manufacturer web page accessed April 2020 excluding shipping costs.

**Table 4:** Bead-beating conditions have a high impact on lysis efficiency of *B. anthracis* spores. Comparison of DNA release from *B. anthracis* spores, as quantified by qPCR after the disruption via bead-beating. Parameters (bead sizes and composition) as well as the homogenizer type on the efficiency of cell disruption are shown.

Sample		Concentration Spores [CFU]	C_t_ Values
	Co	10^8^	27.38 ± 0.17
TL	1 min	10^8^	19.09 ± 0.09
	**5 min**	**10^8^**	**19.02 ± 0.04**
	10 min	10^8^	19.46 ± 0.19
FP	1 min	10^8^	19.09 ± 0.13
	2 × 1 min	10^8^	19.48 ± 0.01
	3 min	10^8^	19.54 ± 0.04
	5 min	10^8^	20.45 ± 0.28
Type of beads	Co	10^6^	35.16 ± 0.55
	Pathogen lysis tubes S	10^6^	28.77 ± 0.20
	Glass beads, 0.5 mm	10^6^	25.92 ± 0.12
	**PowerBead Tubes, 0.1 mm**	**10^6^**	**25.72 ± 0.08**
Amount beads	Co	10^8^	27.60 ± 0.01
	**200 mg**	**10^8^**	**19.03 ± 0.18**
	500 mg	10^8^	19.60 ± 0.23
	1000 mg	10^8^	20.85 ± 0.04
Volume sample	200 µL	10^6^	26.85 ± 0.03
	500 µL	10^6^	26.00 ± 0.08
	**1000 µL**	**10^6^**	**25.98 ± 0.04**

C_t_: cycle threshold. Co: suspension of untreated *B. anthracis* spores. TL: TissueLyser II; FP: FastPrep-24. The optimum settings (used for further experiments in this work) are set in bold type. Data represent the mean (±standard deviation) of three independent experiments, each performed at least in triplicate.

**Table 5:** A combination of mechanical disruption by bead-beating and DNA extraction with the MasterPure kit resulted in the highest DNA extraction efficiency for *B. anthracis* spores. C_t_: cycle threshold.

Sample	Concentration Spores [CFU]	C_t_ Values
Suspension of untreated *B. anthracis* spores (control)	10^6^	34.08 ± 0.01
QIAmp DNA Mini kit	10^6^	28.30 ± 0.23
In-house protocol, based on DNA Investigator kit	10^6^	24.76 ± 1.02
MasterPure kit, lysis supernatant	10^6^	25.99 ± 0.10
MasterPure kit, debris pellet	10^6^	25.40 ± 0.30
In-house protocol, based on DNA Investigator kit	10^3^	36.87 ± 1.10
MasterPure kit (lysis supernatant + debris pellet) *	10^3^	34.01 ± 0.97

* DNA extracted from the debris pellet and DNA isolated from lysis supernatant are each dissolved in 25 µL TE buffer and pooled. Data represent the mean (±standard deviation, SD) of three independent experiments, each performed at least in triplicate.

## Supplementary Materials

The following are available online at https://www.mdpi.com/2076-2607/8/5/763/s1, Table S1. Average DNA yields, qPCR amplification results and ratios 260/280 and 260/230 of samples containing *B. anthracis* and *F. tularensis* cells, obtained using 20 different DNA extraction kits. Figure S1. Exemplary spectrophotometric measurements for four DNA extraction kits. Figure S2. Representative results from gel electrophoresis analysis of 5 µl gDNA from samples containing *B. anthracis* and *F. tularensis* cells extracted with the following commercial kits. Figure S3. Exemplary 5200 Fragment Analyzer results of gDNA extracted from *B. anthracis* cells using standard protocol of MasterPure Complete DNA and RNA Purification kit (black) or the QIAamp DNA Mini kit (red). Figure S4. 5200 Fragment Analyzer results of gDNA extracted from *B. anthracis* cells using standard protocol of MasterPure Complete DNA and RNA Purification kit (A) or with the following modifications: (B) Roti^®^PinkDNA added to the precipitation mixture; (C) glycogen added to the precipitation mixture; (D) glycogen and Roti^®^PinkDNA added to the precipitation mixture. Table S2. Copy number validation by ddPCR. Table S3. Average cell number of *B. anthracis* and *F. tularensis* per aliquot for DNA extraction with the MasterPure Complete DNA and RNA Purification kit and mean C_t_ values obtained with real-time PCR. Table S4. Two DNA extraction methods, (A) the silica-based method (QIAamp DNA Mini kit) and (B) the salting-out method (MasterPure Complete DNA and RNA Purification kit) were compared regarding their ability to deal with an overload of starting material. Figure S5. Read-length vs read-quality plot for sequencing data obtained from two MinION runs. DNA of *B. anthracis* was isolated with the QIAamp DNA Mini kit (A) and the MasterPure Complete DNA and RNA Purification kit (B). Table S5. Similar DNA concentrations can be retrieved from cell debris and from the lysis supernatant.
