# Supplementary material for: Evaluation of a Highly Efficient DNA Extraction Method for Bacillus anthracis Endospores
Source: Microorganisms. 2020 May 20;8(5):763. doi: 10.3390/microorganisms8050763 (PMC7285266; doi:10.3390/microorganisms8050763)
Supplement: Supplementary file 1 [file microorganisms-08-00763-s001.pdf]

Supplementary Materials

Table S1. DNA extraction efficiencies and qualities vary depending on the DNA extraction kit.
Average DNA yields, qPCR amplification results and ratios 260/280 and 260/230 of samples containing *B. anthracis* or *F. tularensis* cells, obtained using 20 different DNA extraction kits. DNA concentration was determined by Qubit 3.0 fluorometer and the quality of extracted DNA was determined using a NanoDrop spectrophotometer. All tests were performed at least in triplicate. The results are expressed as the mean ± standard deviation.

No.	Commercial kit	DNA yield [ng]	*dhp*61 [C_t values]	FraTul [C_t values]	Purity [A260/280]	Purity [A260/230]
1	MasterPure Complete DNA & RNA Purification kit	1001±168	16.46±0.28	12.76±0.22	2.03±0.05	1.97±0.10
2	In-house protocol, based on DNA Investigator kit	683±132	16.55±0.02	13.17±0.15	2.05±0.16	0.16±0.15
3	innu PREP DNA Mini kit	605±19	19.57±0.11	15.45±0.11	1.41±0.29	-1.10±2.30
4	GenElute Bacterial Genomic DNA kit	572±29	18.89±0.24	15.40±0.39	2.12±0.11	1.01±0.05
5	DNeasy Ultra Clean Microbial kit	537±38	16.61±0.19	13.49±0.33	1.90±0.02	-28.83±28.49
6	QIAamp DNA Mini kit	489±69	17.72±0.09	14.51±0.35	2.14±0.02	1.25±0.06
7	NucleoSpin Microbial DNA Mini kit	481±99	17.50±0.26	15.08±0.23	1.61±0.07	-0.41±0.10
8	DNeasy Blood and Tissue kit	477±79	18.85±0.12	16.28±0.09	1.68±0.06	-0.47±0.11
9	MagJet Genomic DNA kit	428±98	17.54±0.31	14.88±0.31	1.94±0.06	-21.43±12.65
10	PureLink Microbiome DNA Purification kit	401±62	18.09±0.27	15.12±0.44	1.91±0.06	-0.73±4.97
11	Wizard Genomic DNA Purification kit	371±51	17.64±0.12	14.48±0.02	1.65±0.08	1.45±0.02
12	QIAamp Cador pathogen Mini kit	204±118	20.03±1.02	16.37±1.82	2.08±0.11	0.09±0.02
13	DNA MiniPrep kit	199±200	23.82±9.73	21.93±10.15	1.40±0.48	-8.22±14.05
14	DNeasy PowerSoil kit	196±31	19.53±0.26	15.65±0.16	1.34±0.02	-0.19±0.04
15	nexttec 1-step DNA isolation kit for Bacteria	187±19	20.54±0.23	16.30±0.22	1.27±0.07	0.43±0.03
16	QIAamp UCP Pathogen Mini kit	184±25	19.35±0.20	16.34±0.14	2.66±0.18	0.09±0.01
17	RTP Bacteria DNA Mini kit	93±1	26.13±0.26	23.26±0.54	3.01±0.46	3.24±2.41
18	smart DNA prep	65±14	20.79±0.37	18.02±0.07	0.41±0.11	-0.01±0.01
19	Echolution Tissue DNA Micro kit	61±40	21.86±1.48	16.55±0.59	1.31±0.07	0.24±0.33
20	QIAmp DNA Microbiome kit	27±1	24.03±0.20	16.32±0.17	2.35±0.47	0.09±0.01

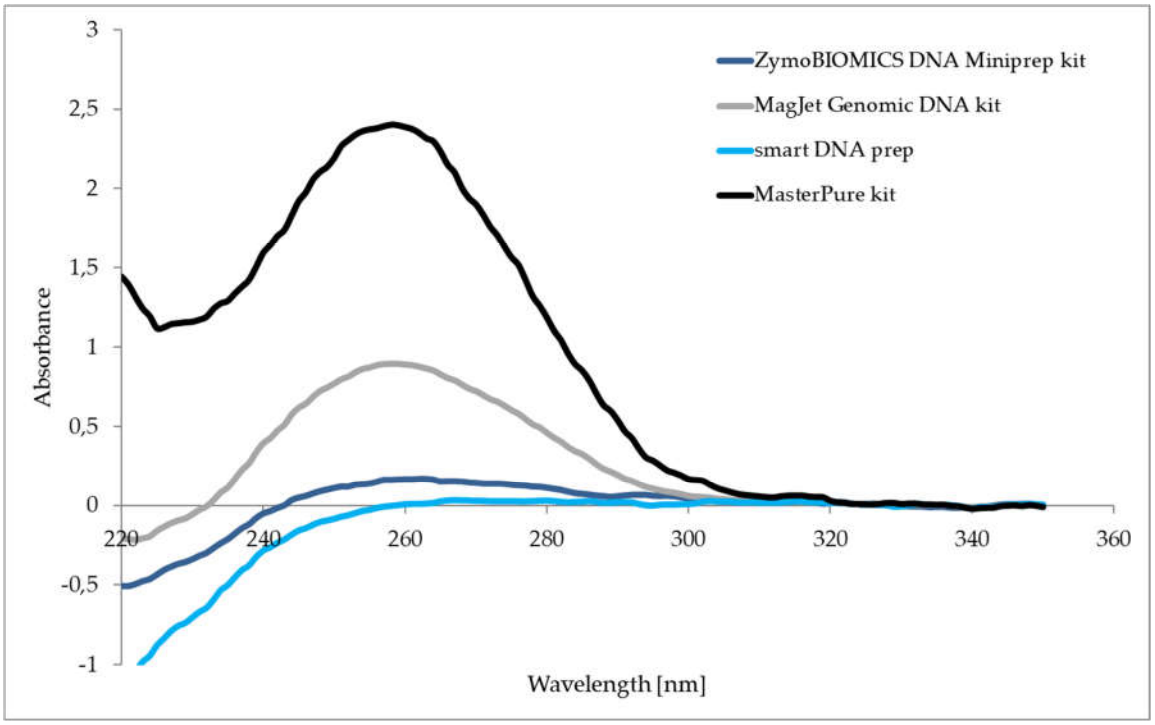

Figure S1. DNA extraction method affects DNA purity.
Exemplary spectrophotometric measurements for four DNA extraction kits are shown, with the highest DNA purity achieved with the MasterPure Complete DNA & RNA Purification kit of Lucigen.

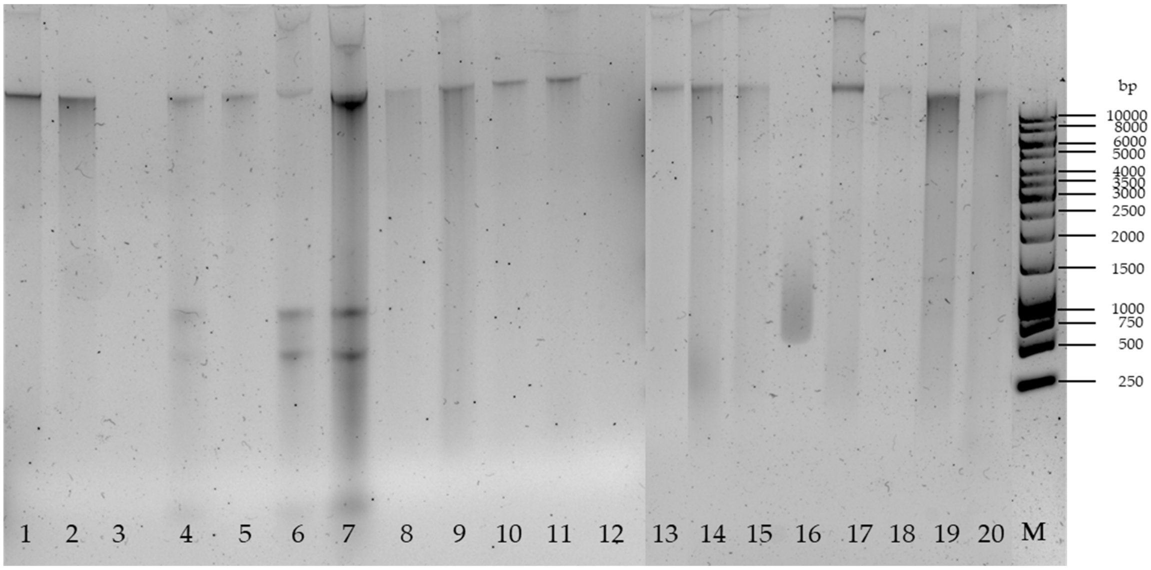

Figure S2. Visualization of DNA integrity of DNA extracted from *B. anthracis* or *F. tularensis* cells by agarose gel electrophoresis.
Representative results from gel electrophoresis analysis of 5 µl gDNA from samples containing *B. anthracis* and *F. tularensis* cells extracted with the following commercial kits: 1) NucleoSpin Microbial DNA; 2) DNA Miniprep kit; 3) smart DNA prep; 4) innu PREP DNA Mini kit; 5) nexttec 1-step DNA isolation kit for Bacteria; 6) MagJet Genomic DNA kit; 7) MasterPure Complete DNA & RNA Purification kit; 8) DNeasy PowerSoil kit; 9) In-house protocol; 10) QIAamp UCP Pathogen Mini kit; 11) DNeasy Blood and Tissue kit; 12) QIAmp DNA Microbiome kit; 13) QIAamp Cador pathogen Mini kit; 14) QIAamp DNA Mini kit; 15) GenElute Bacterial Genomic DNA kit; 16) RTP Bacteria DNA Mini kit; 17) Wizard Genomic DNA Purification kit; 18) Echolution Tissue DNA Micro kit; 19) DNeasy Ultra Clean Microbial kit; 20) PureLink Microbiome DNA Purification kit; M) 1 kb DNA ladder marker.

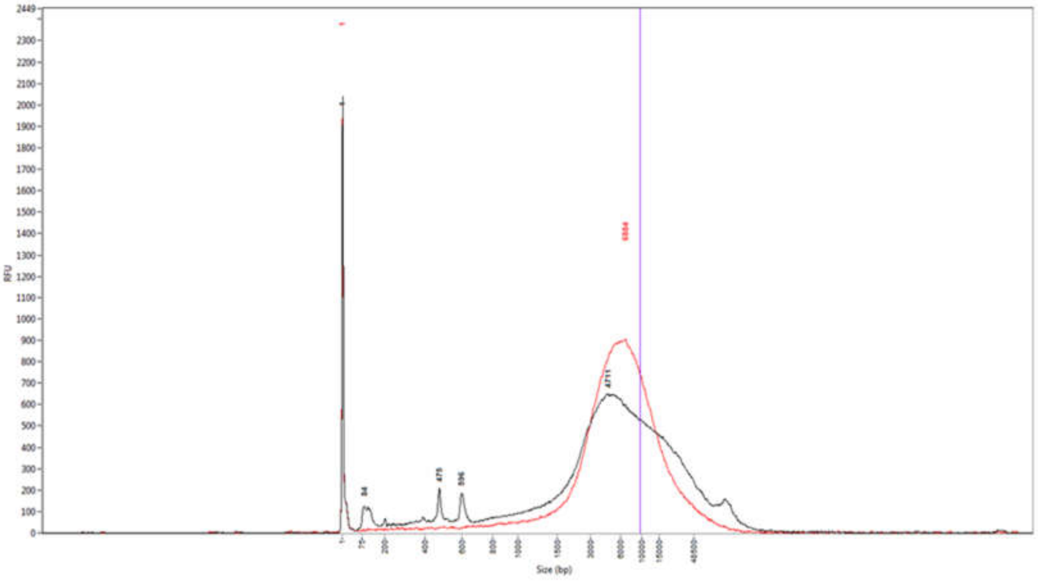

Figure S3. DNA extracted from *B. anthracis* with the MasterPure kit is suitable for nanopore sequencing. Exemplary 5200 Fragment Analyzer results of gDNA extracted from *B. anthracis* cells using standard protocol of MasterPure Complete DNA & RNA Purification kit (black) or the QIAamp DNA Mini kit (red). With the MasterPure kit large amounts of high quality, high molecular weight DNA can be obtained.

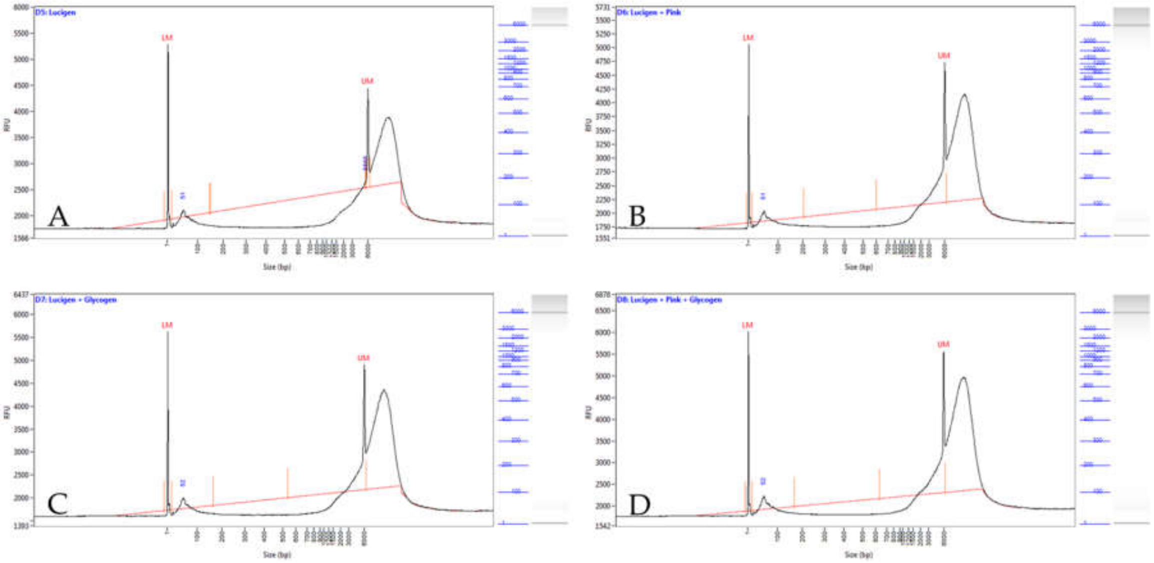

Figure S4. The addition of glycogen and Roti®PinkDNA gives superior DNA yields.
5200 Fragment Analyzer results of gDNA extracted from *B. anthracis* cells using standard protocol of MasterPure Complete DNA & RNA Purification kit (A) or with the following modifications: B) Roti®PinkDNA added to the precipitation mixture; C) glycogen added to the precipitation mixture; D) glycogen and Roti®PinkDNA added to the precipitation mixture.

Table S2. Maximum recovery of genomic DNA of *B. anthracis* and *F. tularensis* is obtained in the presence of glycogen and Roti®PinkDNA.
Copy number validation by ddPCR: The final DNA concentration of DNA extracted from *B. anthracis* cells was validated with ddPCR. DNA was extracted with the standard protocol of the MasterPure Complete DNA & RNA Purification and in the presence or absence of glycogen and/or Roti®PinkDNA. Maximum and minimum Poisson distribution for the 95% confidence interval generated by QuantaSoft™ is also indicated.

Sample	DNA concentration [copies/µL]	PoissonConfMax	PoissonConfMin
Standard protocol	15,981	16,280	15,660
Standard protocol + Roti®PinkDNA	15,659	15,890	15,270
Standard protocol + glycogen	14,855	15,110	14,500
Standard protocol + Roti®PinkDNA + glycogen	21,352	21,770	20,920

Table S3. Detection sensitivity of DNA extraction was improved to 8.6x10^1 cells/mL for *B. anthracis* and 8.6x10^1 cells/mL for *F. tularensis* by modified MasterPure protocol.

Average cell number of *B. anthracis* and *F. tularensis* per aliquot for DNA extraction with the MasterPure Complete DNA & RNA Purification kit and mean C_t values obtained with real-time PCR. All experiments were done at least in triplicate, and the results were reported as mean ± standard deviation.

B. anthracis		*F. tularensis*	
**cells/extraction**	**[C_t values]**	**cells/extraction**	**[C_t values]**
8.6x10^8	11.69±0.00	5.0x10^8	11.04±0.09
8.6x10^7	14.74±0.07	5.0x10^7	13.96±0.06
8.6x10^6	18.83±0.02	5.0x10^6	18.42±0.16
8.6x10^5	23.20±0.22	5.0x10^5	22.20±0.11
8.6x10^4	27.41±0.34	5.0x10^4	25.55±0.12
8.6x10^3	31.64±0.19	5.0x10^3	29.23±0.16
8.6x10^2	35.06±0.40	5.0x10^2	32.19±0.47
8.6x10^1	37.31±1.40	5.0x10^1	36.85±0.17

Table S4. The improved DNA extraction protocol is highly efficient and robust for DNA overload and PCR inhibitors.

Two DNA extraction methods, A) the silica-based method (QIAamp DNA Mini kit) and B) the salting-out method (MasterPure Complete DNA & RNA Purification kit) were compared regarding their ability to deal with an overload of starting material. Therefore, 1 mL of sheep blood and 1 mL of an *E. coli* overnight culture (approximately 10^{10} cells) were spiked with 10^8 cells of *B. anthracis*. The DNA extracted was eluted or dissolved in 200 µL TE buffer, respectively. DNA concentrations were measured with the Qubit Fluorometer using the dsDNA HS assay kit. The amount of *B. anthracis* cells was determined via qPCR. Averaged C_t values and DNA concentratins (from triplicates) and standard deviations (SD) are calculated.

Kit	Sheep blood		*E. coli* overnight culture	
	**[C_t values]**	**DNA concentration [ng/µL]**	**[C_t values]**	**DNA concentration [ng/µL]**
A	29.20±2.21	1.19±0.13	18.84±0.13	17.25±2.04
B	21.29±0.37	21.65±1.38	14.60±0.17	303.00±51.14

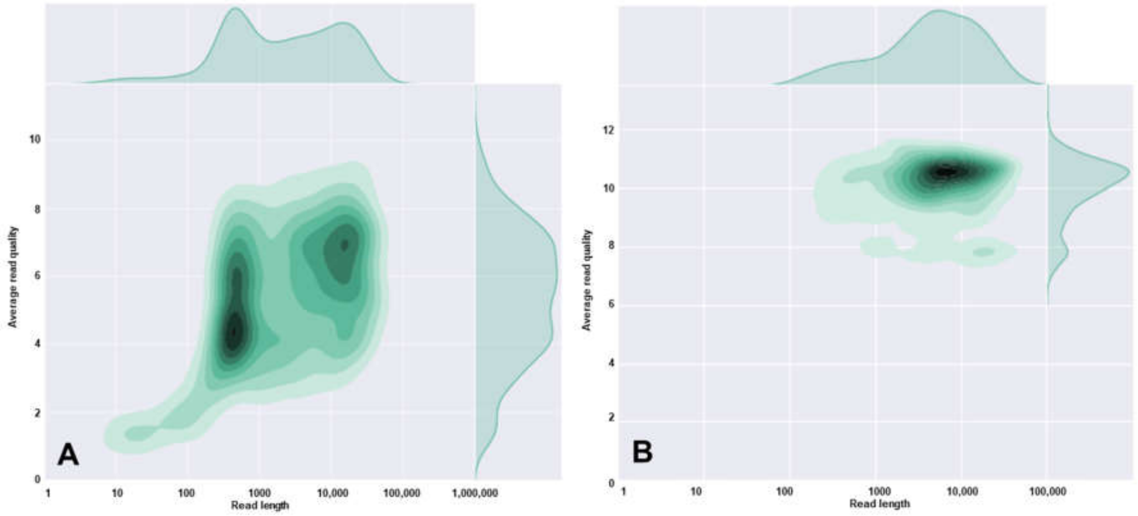

Figure S5. DNA extraction method impacts the quality of sequencing results.
Read-length vs read-quality plot for sequencing data obtained from two MinION runs. DNA of *B. anthracis* was isolated with the QIAamp DNA Mini kit (A) and the MasterPure Complete DNA & RNA Purification kit (B). Library preparation was conducted according to the manufacturers' protocol, using the Native Barcoding expansion pack (EXP-NBD104) and the 1D Sequencing kit, with the SQK-LSK109 chemistry. No additional shearing was performed.

Table S5. Similar DNA concentrations can be retrieved from cell debris and from the lysis supernatants. Copy number validation by ddPCR: The final DNA concentration of DNA extracted from *B. anthracis* spores was validated with ddPCR. After bead-beating DNA was extracted from the debris pellet and from lysis supernatant with the MasterPure Complete DNA & RNA Purification kit. Poisson distribution for the 95% confidence interval generated by QuantaSoft™ is also indicated.

**Sample**	**Concentration spores [CFU]**	**DNA concentration [copies/µL]**	**PoissonConfMax**	**PoissonConfMin**
Cell debris	10^6	2645	2950	2340
Lysis supernatant	10^6	2630	2930	2330
Cell debris	10^5	265	2950	2350
Lysis supernatant	10^5	278	3100	2460
